# *Francisella *infections in farmed and wild aquatic organisms

**DOI:** 10.1186/1297-9716-42-47

**Published:** 2011-03-08

**Authors:** Duncan J Colquhoun, Samuel Duodu

**Affiliations:** 1 Section for Fish health, National Veterinary Institute, Postbox 750 sentrum, 0106 Oslo, Norway

## Abstract

Over the last 10 years or so, infections caused by bacteria belonging to a particular branch of the genus *Francisella *have become increasingly recognised in farmed fish and molluscs worldwide. While the increasing incidence of diagnoses may in part be due to the development and widespread availability of molecular detection techniques, the domestication of new organisms has undoubtedly instigated emergence of clinical disease in some species. Francisellosis in fish develops in a similar fashion independent of host species and is commonly characterised by the presence of multi-organ granuloma and high morbidity, with varying associated mortality levels. A number of fish species are affected including Atlantic cod, *Gadus morhua*; tilapia, *Oreochromis *sp.; Atlantic salmon, *Salmo salar*; hybrid striped bass, *Morone chrysops *× *M. saxatilis *and three-lined grunt, *Parapristipoma trilinineatum*. The disease is highly infectious and often prevalent in affected stocks. Most, if not all strains isolated from teleost fish belong to either *F. noatunensis subsp. orientalis *in warm water fish species or *Francisella noatunensis *subsp. *noatunensis *in coldwater fish species. The disease is quite readily diagnosed following histological examination and identification of the aetiological bacterium by culture on cysteine rich media or PCR. The available evidence may indicate a degree of host specificity for the various *Francisella *strains, although this area requires further study. No effective vaccine is currently available. Investigation of the virulence mechanisms and host response shows similarity to those known from *Francisella tularensis *infection in mammals. However, no evidence exists for zoonotic potential amongst the fish pathogenic *Francisella*.

## 1. Introduction

As the aquaculture industry worldwide intensifies and diversifies, it is natural that domestication of new aquaculture species results in recognition of "new" infectious agents and diseases. This has been demonstrated repeatedly over the years. In recent years bacteria belonging to the genus *Francisella *have "emerged" as serious pathogens of various fish species, both farmed and wild, from various geographical regions worldwide [1-7]. The most recent addition to the list represents the first isolation of a molluscan pathogenic *Francisella *sp. [8]. Francisellosis associated with aquatic organisms is probably not truly novel. The recent spate of diagnoses may be partially related to the increased awareness of such infections combined with adoption of suitable culture media and the widespread availability of non-culture based molecular detection techniques. However, and for whatever reason, it is clear that *Francisella *infections in fish are serious and more widely distributed than previously thought just a few years ago. Given the relative recent nature of the discovery of these diseases, much scientific work is currently in progress and many research results remain as yet unpublished. While the present review will restrict reporting of research results in the main to published work, as a measure of necessity, references to unpublished work, manuscripts in preparation and personal communications are occasionally made.

## 2. *Francisella *taxonomy and nomenclature

**Figure 1:**
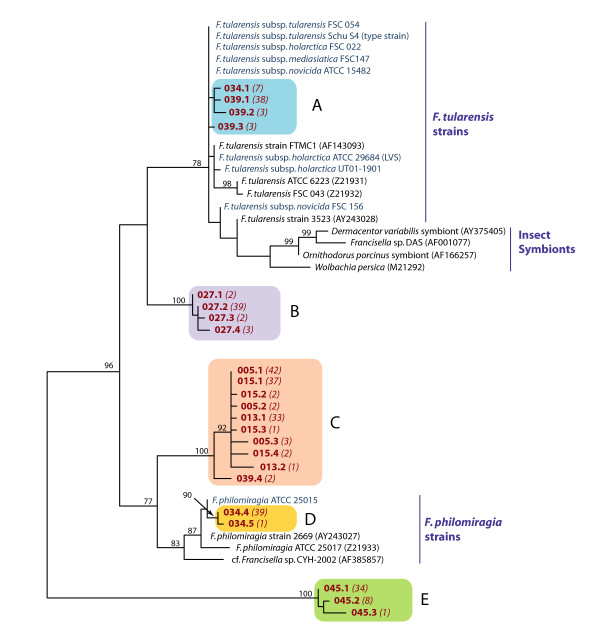
**Phylogenetic tree showing the two major lineages of *Francisella *inferred from the 16S rDNA sequences of reference strains and environmental samples**. Taken from Barns et al. [11].

The genus *Francisella *consists of non-motile, Gram-negative, strictly aerobic, facultatively intracellular cocco-bacilli and currently includes four validly published species. The type species of the genus is the agent of tularemia, *F. tularensis *[9], a highly infectious bacterium causing disease in mammals including humans and a potential bio-terror weapon. Although until very recently the validly published members of the genus *Francisella *could be divided into two major lineages on the basis of phylogenetic analysis of the 16S rRNA gene (Figure 1), i.e. the *F. tularensis *lineage and the *F. philomiragia *lineage, a third lineage, comprising *F. hispaniensis *as the sole member has, been recently described [10]. Molecular studies of environmental samples have also demonstrated the existence of as yet undescribed members of this genus [11,12]. The taxonomic situation within the genus *Francisella *is complex and currently relatively dynamic. While *F. novicida *has been very recently reclassified as a subspecies of *F. tularensis *[10] i.e. *F. tularensis *subsp. *novicida*, prior to description of the first fish pathogenic species[13], the genus contained three species i.e. *F. tularensis *[14], *F. philomiragia *[15] and *F. novicida *[16]. The situation became less clear as the description of various isolates from various fish species began.

### 2.1. The fish pathogenic Francisella: nomenclature

**Figure 2:**
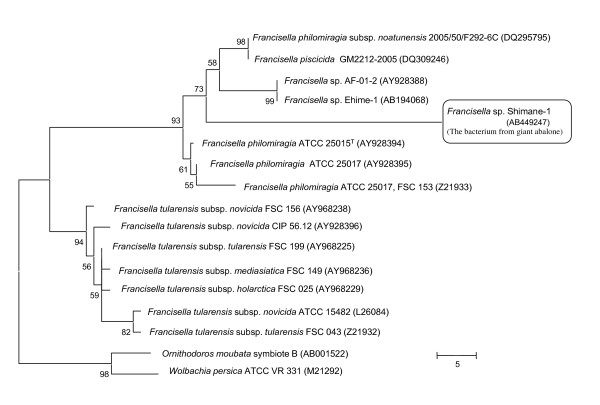
**Phylogenetic relationship of the *Francisella *sp. bacterium isolated from a diseased giant abalone *Haliotis gigantea*, inferred from the 16S rDNA sequences**. Taken from Kamaishi et al. [3].

Molecular characterization of the 16S rRNA gene has demonstrated the existence of two different genetic lineages among the fish pathogenic *Francisella *isolates, with a single mollusc pathogenic strain belonging to a separate (with a long branch length indicating a considerable time since they shared a common ancestor) branch of the same clade (Figure 2). All three lineages are closely related to the opportunistic human pathogen *F. philomiragia *[2,13]. Of the two fish pathogenic lineages, Mikalsen et al. [13] proposed the seven isolates examined from diseased Atlantic cod from Norway to represent a subspecies of *F. philomiragia *i.e. *F. philomiragia *subsp. *noatunensis*. Shortly after, Ottem et al. [17] proposed establishment of a new species, *F. piscicida*, based on examination of a single isolate from diseased Atlantic cod. As 16S rDNA sequences for *F. philomiragia *subsp. *noatunensis *and *F. piscicida *were 100% similar it was considered that they may represent heterotypic synonyms [18]. Following comparison of the two type strains this was subsequently proven to be the case [19,20], with both [19] and [20] proposing elevation of *F. philomiragia *subsp. *noatunensis *to *F. noatunensis*. In separate studies, *Francisella *strains (including a strain common to both studies) isolated from tilapia and three-lined grunt were proposed to represent 1) a subspecies of *F. noatunensis *(subsp. *orientalis*) [20] and 2) an independent species, *F. asiatica *[19]. The latter proposal, published online by The International Journal of Systemic and Evolutionary Microbiology, cannot however, according to the International Code of Nomenclature of Prokaryotes (due to the rule on prior publication [20]) be considered validly published and should therefore not be used. The single *Francisella *isolate examined by Mikalsen et al. [19] from diseased salmon farmed in fresh water in Chile [1] should therefore be considered a strain of *F. noatunensis *subsp. *noatunensis*. While Kay et al. [21] referred to a *Francisella *isolated from tilapia as "*F. victoria"*, this name has not been validly published and cannot be correctly used. The current validly published members of the genus *Francisella *are listed in Table 1.

**Table 1:** Validly published species of the *Francisella *genus.

*Francisella tularensis *lineage	*Francisella philomiragia *lineage
*Francisella tularensis *subsp. *tularensis*	*Francisella philomiragia*

*Francisella tularensis *subsp. *holarctica*	*Francisella noatunensis *subsp. *noatunensis*

*Francisella tularensis *subsp. *mediasiatica*	*Francisella noatunensis *subsp. *orientalis*

*Francisella tularensis *subsp. *novicida*	

***Lineage "3"***	

*Francisella hispaniensis*	

### 2.2. The diversity of as yet undescribed Francisella

Although culture of *Francisella *from the environment is possible [12,22], it is notoriously difficult. Recent studies of fish microbiota [23] and environmental samples [11,12] utilising non-culture based methodology have, however, clearly revealed the existence of significant numbers of as-yet undescribed *Francisella *and *Francisella*-related species associated with fish and the environment. A number of gene sequences retrieved from these samples belong to the *F. philomiragia lineage *and are therefore closely related to currently known fish pathogenic species. An as yet un-cultured endosymbiont of the ciliate *Euplotes raikovi *has been proposed as a novel subspecies of *F. noatunensis *i.e. *Candidatus *F. noatunensis subsp. endociliophora [24], but this name has not yet been validly published according to the International Code of Nomenclature of Prokaryotes. As nearly all such environmental detections to date have been restricted to aquatic environments, these studies may give some indication of the battery of possible "pathogens" awaiting new aquaculture species.

## 3. The disease/s

Systemic infections in fish caused by Gram-negative intracellular bacteria refractive to culture on standard laboratory media have been recognized for many years. Such infections have been commonly referred to as either *Rickettsia*-like (RLO) due to morphological similarities with the true *Rickettsia *or *Piscirickettsia*-like organisms (PLO) following the description of *Piscirickettsia salmonis *[25]. The genus *Francisella *is in fact relatively closely related and similar both morphologically and in terms of pathogenesis, to *Piscrickettsia salmonis*. However, as the latter organism and its diseases have been extensively reviewed [26,27], this genus will not be covered in the present review beyond mention here of two recent and significant developments in *Piscirickettsia *research i.e. the discovery that this bacterium has a facultatively (not obligatory, as previously considered) intracellular nature and may in fact be cultured on cysteine enriched agar media [28,29], along with the apparent emergence of a novel *Piscirickettsia *species causing disease in muskellunge, *Esox masquinongy *and yellow perch, *Perca flavescens *[30]. Despite morphological similarities, the genera *Francisella *and *Piscirickettsia *belong to the γ-proteobacteria and are therefore only distantly related to the true *Rickettsia *(*α-*proteobacteria).

While the "agent of tularemia" presumably *F. tularensis*, was related to infections in fish as early as 1970, this bacterium has not been associated with fish disease in later years [31]. In light of the recent description of the fish pathogenic species, which share a number of phenotypic traits with *F. tularensis*, it might be speculated that these early detections may have been a case of misidentification. An outbreak of water borne tularemia associated with crayfish fishing in Spain could not be attributed to the crayfish themselves [32]. The "modern" emergence of francisellosis probably started with the identification of a *Rickettsia*-like organism (RLO) in diseased tilapia farmed in both fresh and saltwater in Taiwan [33], which is probably the *Francisella*-like organism described in Taiwanese tilapia by Hsieh et al. [2]. Francisellosis was subsequently identified in farmed tilapia in Latin America [5], more specifically Costa Rica [19,34] and several states in mainland USA [4], while a similar disease associated with a PLO in farmed tilapia in Hawaii [35], is as yet unconfirmed as francisellosis. The bacterium has additionally been isolated from tilapia in Indonesia [20] and recently confirmed in tilapia farmed in recirculated systems in England [36]. Other species affected include hybrid striped bass, *Morone chrysops *x *M. saxatilis *in california [37] and three-lined grunt, *Parapristipoma trilinineatum *in Japan (imported from China) [38]. Other RLO infections which could conceivably be related to *Francisella *spp. include the RLOs reported from ornamental blue-eyed plecostamus, *Panaque suttoni *[39] and dragonet, *Callionymus lyra *[40]. However, it should not be assumed that all RLO/PLO are in fact *Francisella *spp. The *Piscirickettsia salmonis*-like organism reported from cultured grouper, *Epinephelus melanostigma *in Taiwan [41], in contrast to the confirmed *Francisella *infecting tilapia [5] reacted positively with polyclonal anti-*P. salmonis *sera and may therefore be more related to *Piscirickettsia *than *Francisella*.

**Figure 3:**
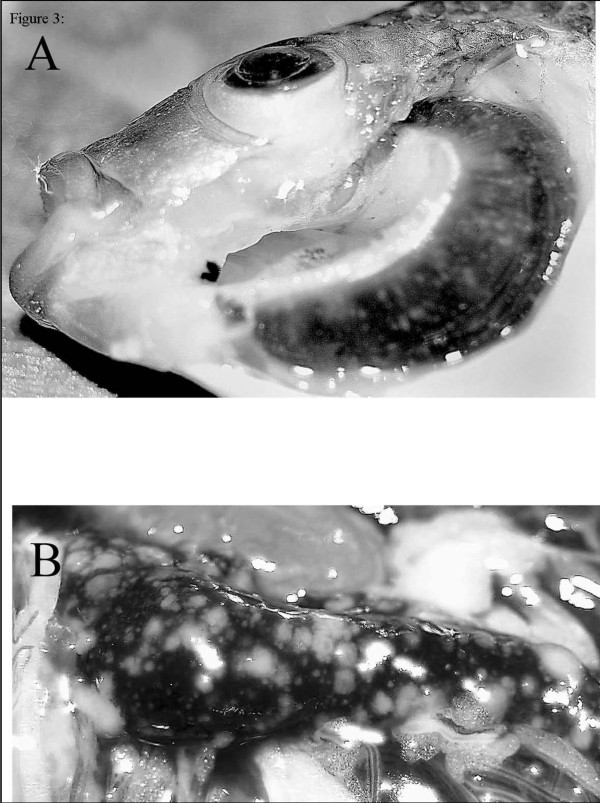
**South Carolina tilapia with the gross signs of the severe, chronic stage of the PLO disease**. Granulomas in the gills **A**, and in the spleen **B**. Taken from Mauel et al. [4].

All described incidences of francisellosis in fish manifest in a similar fashion which can be summarised as systemic, chronic, granulomatous infections resulting in varying degrees of mortality. Common observations in Atlantic cod, three-lined grunt, tilapia, hybrid striped bass and ornamental cichlids include the extensive occurrence of white, partly protruding nodules (granuloma) of various size in the spleen (Figure 3), kidney and liver [2,3,7,37,42]. Other organs which may be affected include virtually any tissue type, as associated pathological changes have also been described in the gill (Figure 3) heart, testes, musculature, brain and eye. In Atlantic cod, the spleen is generally enlarged and sero-haemorrhagic ascites and thickened intestinal mucosa may be observed. Extensive chronic granulomatous inflammation with multiple granuloma in all organs is the main histopathological finding with few to numerous, small Gram-negative bacteria, sometimes observed within granuloma [7]. A granulomatous condition is also reported in association with *Francisella *infections in Atlantic salmon [1,43]. No granuloma were observed in association with the first report of Francisellosis in a non-vertebrate i.e. abalone [8]. Although the strain of bacterium involved in the abalone disease clusters phylogenetically with the fish pathogenic clade, it is the most phylogenetically distant member of that clade. The lack of granuloma formation in abalone may be more related to the molluscan immunological repertoire, rather than differences in the infecting bacterium. Reported mortalities associated with natural infections range from low level in striped bass [37], 5-20% in Atlantic salmon [43] to 95% in tilapia [33]. Francisellosis in cod is associated with varying mortality levels. While mortalities of up to 40% have been described [7], such levels of relatively acute mortality are rarely experienced in the field. Environmental conditions, in particular temperature, appear to play a significant role in the rate of mortality. On examination of the literature it is important to note that temperatures considered low for tilapia culture, exceed the maximum temperature at which coldwater species such as cod, may be cultured. Generally, the higher the temperature, the more acute the disease is likely to be, with mortality levels increasing with temperature until a maximum pathogenic temperature is reached [8,37]. The presence of mixed infections with other fish pathogenic bacteria may also significantly affect mortality rates.

### 3.1. Differential diagnoses

Several bacterial diseases may present in a similar manner to francisellosis. Piscine mycobacteriosis, commonly characterised by macroscopically visible multi-organ granuloma caused by a diverse range of different *Mycobacterium *spp. has been identified in a large number of cultured and wild fish species around the world [44]. Mycobacteria can be refractive to culture and are not always readily observable in histological preparations even when Ziehl-Neelson stained. *Nocardia *spp. infections may also present in a granulomatous form [45]. *Piscirickettsia salmonis *infections, which may also present in a similar fashion to francisellosis is most commonly associated with salmonid fish species, yet has been identified in an increasingly diverse range of fish species e.g. European seabass, *Dicentrachus labrax *[46] and white seabass, *Atractoscion nobilis *[47]. One of the most common systemic bacterial infections affecting populations of farmed cod in Norway is atypical furunculosis [48], caused by atypical isolates of *Aeromonas salmonicida*. This type of infection can result in a disease presenting macroscopically very similar to francisellosis. Although both diseases result in extensive granuloma development, they are quite readily differentiated by histological examination. Mixed infections with *F. noatunensis *and atypical *A. salmonicida *and/or *Vibrio anguillarum *are also relatively common [49].

### 3.2. Farmed vs. wild fish

Many systemic bacterial diseases result in relatively rapid death of the affected fish, which disappear from the population and are therefore difficult to detect at low prevalence in wild fish populations. The chronic nature and lengthy course of francisellosis, particularly in cold water marine species such as cod, probably mean that the likelihood of detection of francisellosis in wild fish is more likely than with other Gram-negative infections. Francisellosis is, however, a relatively recently recognised disease, and reports from wild fish are as yet relatively rare. A prevalence of approximately 20%, based on macroscopic observations, was identified in a single year class of wild cod captured off the Swedish west coast in 2004 [50]. Farmed Atlantic cod in Norway are held in net cages in close contact with wild fish (including wild Atlantic cod) which congregate around these structures. A recent screening [51] of farmed and wild cod as well as other species of fish caught around the Norwegian coastline using Real Time PCR, reported the relatively widespread presence of *F. piscicida *(a.k.a. *F. noatunensis*) in wild cod (prevalence 7-11%), from both areas with and without cod farms, although fish exhibiting clinical signs of disease were rare. Unfortunately the disease/infection status in wild fish prior to recent outbreaks in farmed cod is not known and little is understood of the effect of infection pressure from farmed fish to wild fish in these areas. Low levels of infection were also identified in several marine fish species i.e. coalfish, *Pollachius virens*, pollock, *Pollachius pollachius*, mackerel, *Scomber scombrus*, European plaice, *Pleuronectes platessa *and megrim, *Lepidorhombus whiffiagonis *and other aquatic organisms such as blue mussels, *Mytilus edulis *and edible crab, *Cancer pagurus*. However, the significance of these low level detections is difficult to estimate, considering the extreme sensitivity of the assay and that samples were collected mainly in the proximity of affected cod farms. A low prevalence of PCR positive fish in populations of migratory cod (spawning migration from the Barents Sea) caught off the Lofoten archipeligo in Northern Norway has also been reported [52]. That clinical francisellosis is a disease of long standing in nature has been established in a retrospective study utilising paraffin-embedded samples performed in our own laboratory, which confirmed the existence of francisellosis in wild cod in the North Sea during the 1980s [53]. There are no published reports of francisellosis caused by *F. noatunensis *subsp. *orientalis *in wild fish.

### 3.3. Host specificity

Little information is available relating to specificity of the various *Francisella *species for the various species of fish from which they are most commonly isolated. *F. noatunensis *subsp. *orientalis *(or very closely related bacteria), most commonly isolated from tilapia does, however, cause disease in a number of other fish species including three-line grunt [38] and a variety of ornamental cichlids [42], while experimental infections following intraperitoneal injection of *F. noatunensis *subsp. *orientalis *have been established in red sea bream, *Pagrus major *[8] and zebrafish, *Danio rerio *[54]. That a dose equivalent to 23 cfu was capable of causing mortality in tilapia [55] while a much higher dose of 3.45 × 10^5 ^cfu was required to cause very low mortality in zebrafish [54] indicates a degree of host specificity at least under the experimental conditions. The virulence of *F. noatunensis *subsp. *noatunensis *isolated from Atlantic salmon [1] and cod for other species of fish is as yet untested or at least undescribed in the literature. Although the total numbers of wild fish other than cod studied by Ottem et al. [51], were low, generally higher numbers of *F. noatunensis *were identified in wild cod than non-cod species. Ottem et al. [51] also reported finding significant levels of *F. noatunensis *subsp. *noatunensis *in one farmed Atlantic salmon by quantitative PCR, yet no clinical sign of disease in salmon has been identified in Norway, despite regular surveillance in large, dense populations of salmon farmed in the immediate vicinity of infected cod populations. This, together with the fact that only cod were identified displaying clinical signs of disease in the Swedish epizootic [50], may indicate that the north Atlantic strain of *F. noatunenis *subsp. *noatunensis *has an affinity for Atlantic cod greater than for other species of fish. The *Francisella *sp. pathogenic for giant abalone, *Haliotis gigantea *described by Kamaishi et al. [8] while also virulent in the Japanese black abalone, *Haliotis discus discus *and identified as the presumptive agent of disease in Yezo abolone, *Haliotis discuss hannai*, is apparently unable to cause disease in the teleost red seabream.

### 3.4. Zoonotic potential

While there is some strain dependent variation, *F. tularensis *is widely recognised as a highly virulent zoonotic agent. *F. philomiragia*, with which the fish pathogenic species are relatively closely related, also poses a slight, but real zoonotic potential, particularly in individuals with suppressed immunity [56-58]. While both *F. tularensis *and *F. philomiragia *are capable of laboratory growth at 37°C, none of the fish pathogenic species are capable of growth at this temperature. Mikalsen et al. [59] tested pathogenicity of *F. noatunensis *subsp. *noatunensis *and *F. noatunensis *subsp. *orientalis *in mice by intraperitoneal injection of relatively high doses of bacteria (5-7 × 10^7 ^cfu), without any adverse reaction, disease or subsequent re-isolation of bacteria from internal organs. Thus, laboratory-based evidence would suggest that it is highly unlikely that fish pathogenic *Francisella *pose a risk of zoonotic infection. It is probably relevant in this context to consider *F. noatunensis *subsp. *orientalis *more closely. Of the fish pathogenic *Francisella *species described to date, this bacterium has the highest optimal and maximum growth temperature and has most commonly been identified in tilapia around the world. Tilapia possess fin spikes which often cause skin injury during handling and/or preparation and such skin injuries may be associated with transmission of zoonotic infections e.g. *Streptococcus iniae *[60]. That many hundreds of thousands of tilapia infected with *F. noatunensis *subsp. *orientalis *must have been handled, processed, prepared and eaten during the last decade/s, without a single case of associated disease being reported, probably constitutes the most compelling "evidence" for lack of zoonotic capability in this group of bacteria.

## 4. Transmission and environmental survival

Members of the *Francisella *genus are non-motile and are "transmitted by direct contact with infected animals, through contaminated water or food, or by vectors such as biting insects" [9]. Transmission of francisellosis in fish has an obvious connection with the aquatic environment, and the disease has been identified in both fresh and marine waters [1,3,7,13,38]. It would appear that francisellosis is highly transmissible under optimal environmental conditions as prevalence of infection within affected stocks of farmed Atlantic cod and tilapia can be extremely high [7,33] although there is some evidence (Colquhoun, unpublished results) that francisellosis transmission in cod may be reduced at lower temperatures. Tularemia i.e. *F. tularensis *is known to have a very small least infectious dose of 10 bacterial cells or less [61]. This trait appears to be shared with fish and mollusc pathogenic *Francisella*, as few as 1 - 23 cfu *F. noatunensis *subsp. *orientalis *injected intraperitoneally were capable of causing disease in tilapia while 32 cfu of the abalone pathogenic *Francisella *sp. described by Kamaishi et al. [8] killed 100% of intramuscularly injected abolone within 16 days of infection. While the minimum infectious dose for *F. noatunensis *subsp. *noatunensis *in cod has not been established, laboratory trials have confirmed the rapid transmission and chronic course of disease in cod [6,59]. Fish to fish contact is unnecessary and cod may be directly infected via effluent water from tanks containing infected fish (M. Schrøder, pers. comm.). In a cohabitant challenge performed at 12°C, all cohabitant Atlantic cod sampled after 38 days were infected [59] and by the end of the five month cohabitation period, 100% of cohabitant fish displayed severe macroscopic signs of disease and were culture positive. Interestingly few fish died during this period. Not surprisingly, water temperature appears to play a significant role in development of francisellosis. Progression of infection, transmission and mortality associated with *F. noatunensis *subsp. *noatunensis *in cod is low at the lower end of water temperature at which cod may be farmed (< 4°C), although bacteria may also be readily cultured from infected fish during the winter months (Colquhoun, pers. obsv.). The course of disease increases with water temperature up to the maximum temperature at which cod may survive (approaching 20°C). Infection and transmission of *F. noatunensis *subsp. *orientalis *appears restricted to 20-28°C in hybrid striped bass [37] and greater mortality was identified at 15°C than at 30°C in tilapia [33]. Salinity does not seem to have a significant role in disease development as *F. noatunensis *subsp. *noatunensis *has been identified in marine farmed Atlantic cod [7] and in Atlantic salmon farmed in freshwater [1], while *F. noatunensis *subsp. *orientalis *has been isolated from hybrid striped bass and tilapia in fresh water [37,62] and three-lined grunt in seawater [3,38]. In the previously mentioned laboratory trial, *F. noatunensis *subsp. *noatunensis *could be cultured from the gut of 50% of cohabitant Atlantic cod at termination of the trial [59], which may indicate the fecal-oral route as an important route of transmission. Identification of *F. noatunensis *subsp. *noatunensis *in Atlantic cod eggs may also indicate that the disease can be transmitted vertically, although, this needs to further examined [63]. There is evidence that *F. tularensis *persist in a viable but non-cultivable (VBNC) state in cold water [64]. Duodu and Colquhoun [65] found *F. noatunensis *to enter the same state after 30 and 16 days at 8°C and 12°C, respectively. Although metabolically active, the VBNC fish pathogenic *Francisella *(in common with *F. tularensis*) were non-virulent, at least under the experimental conditions tested. It may be that the conditions for revival of virulence were simply not met. A reservoir in aquatic protozoans has been proposed [66].

## 5. Diagnosis and detection of *Francisella *infections

### 5.1. Macroscopic examination

While severely affected populations often show a high rate of morbidity, from field experiences in Norway it is clear that the disease may become highly prevalent prior to noticeable change in fish appearance or behaviour. Initial clinical signs (in severely affected fish) include emaciation, dark colouration and raised haemorrhagic nodules [6,7] or skin ulceration [5] may be observed. Internal macroscopically visible changes are dominated by the multi-organ granuloma development described previously.

### 5.2. Histological examination

Histological examination of formalin-fixed paraffin embedded tissues (FFPE) is one of the most commonly used diagnostic procedures in fish disease investigation. The histological picture, at least for those species of affected fish for which histological investigations are described, appear to be similar [2,4,5,7,34,38,55]. They show extensive granulomatous inflammation with multiple granulomas [7], many of which may be liquid-filled [6]. Cells within the granuloma are dominated by hypertrophied foamy macrophages [5,7], fibroblasts and leukocytes [6]. Granulomas may display a necrotic core [5,6]. Focal to diffuse necrosis and necrotising vasculitis in affected organs, accompanied by infiltration of mononuclear cells and granuloma formation were described by Mauel et al. [5]. Few or no bacteria may be observable particularly in cases of advanced disease with extensive mature granuloma [6]. Such lesions may be observed in almost any organ or tissue type including the meninges in severe infections [5].

### 5.3. Culture

The gold standard for diagnosis of francisellosis, in common with any other systemic bacterial disease in fish, is culture of the bacterium in question combined with macroscopic and histological observations consistent with the disease. Members of the genus *Francisella *are generally fastidious in their requirements for growth on laboratory media and most, including all fish pathogenic strains isolated to date, have a common requirement for the amino acid cysteine. Various media types have been used for primary isolation of *Francisella *spp. from fish (Table 2) all of which include elevated levels of cysteine (or cystine) and glucose. It is pertinent to point out that these bacteria cannot be cultured on the routine, general purpose agar types normally used in general fish diagnostic bacteriology e.g. tryptone soya agar, heart infusion agar or blood agar without additional cysteine. For long term storage of fish pathogenic *Francisella*, lyophilisation is probably the best option, although reports from culture collections indicate that successful lyophilisation is not without challenge. In our laboratory we have successfully maintained *Francisella *(both *F. noatunensis *subsp. *noatunensis *and subsp. *orientalis*) stock cultures over several years at -80°C in a general purpose broth medium (without additional cysteine) containing 15-20% glycerol.

**Table 2:** Media used for isolation of *Francisella *spp. from fish.

Bacterium	Fish species	Media type	Reference
*Francisella noatunensis *subsp. *noatunensis*	Atlantic cod *Salmo salar*	cysteine heart agar + 5% ovine blood	Olsen et al. [7]

*Francisella noatunensis *subsp. *noatunensis*	Atlantic salmon *Salmo salar*	cysteine heart agar + 5% ovine blood	Birkbeck et al. [1]

*Francisella noatunensis *subsp. *orientalis*	Tilapia *Oreochromis *sp.	cysteine heart agar + 5% ovine blood	Mikalsen et al. [19]^#^

*Francisella noatunensis *subsp. *orientalis*	Three-lined grunt *Parapristipoma trilinineatum*	cysteine heart agar + 1% haemoglobin	Kamaishi et al. [3]

*Francisella *sp.¤	Tilapia *Oreochromis *sp.	Thayer-Martin agar	Hsieh et al. [2]

*Francisella *sp.¤	Tilapia *Oreochromis *sp.	modified Thayer-Martin agar, selective cysteine heart agar + bovine haemoglobin, selective cystein heart agar + rabbit blood	Soto et al. [34]

# although not specifically stated as primary isolation medium, this is documented.

¤ 100%16S rRNA gene sequence identity with *Francisella noatunensis *subsp. *orientalis*.

#### 5.3.1. Selective agar media

**Figure 4:**
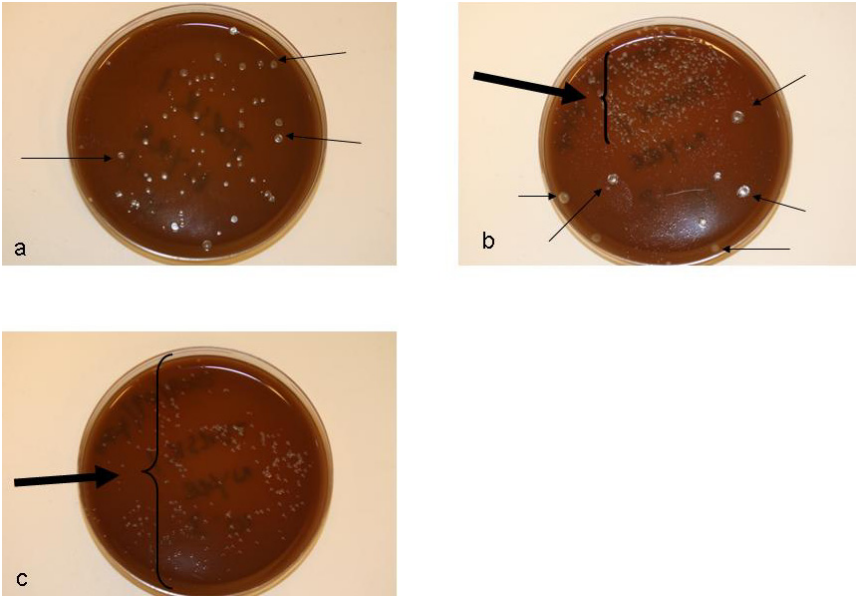
**Inhibition of *Francisella noatunensis *subsp. *noatunensis *by contaminating bacteria in spleen homogenate; a) 10^-1 ^dilution; b) 10^-2 ^dilution; c) 10^-3 ^dilution thin arrow = contaminants thick arrow = *F. noatunensis *bacteria**.

Experiences in our own laboratory confirm that isolation of *F. noatunensis *subsp. *noatunensis *from Atlantic cod is readily inhibited by growth of a wide range of bacteria, both fish pathogenic and environmental (Figure 4). This is a particularly relevant problem in diagnostic work, due to the common presence of mixed infections/infiltration of environmental bacteria. Such mixed infections are most probably due to the chronic nature of francisellosis and consequential weakening of the immune system in affected fish. Several agars selective for *Francisella *species have been published, including a cysteine heart agar containing colistin, amphotericin, lincomycin, trimethoprim and ampicillin for selective culture of *Francisella tularensis *[22,67]. The same agar, but excluding amphotericin was successfully used to isolate *F. philomiragia *from environmental material by Berrada and Telford [12]. While we have not been able to culture either *Francisella noatunensis- *subsp. *noatunensis *or- subsp. *orientalis *on the selective agar described by Petersen et al. [22] in our own laboratory, selective agars containing polymixin B with- and without- ampicillin were used successfully for isolation of *Francisella noatunensis *subsp. *orientalis *(putatively) by Soto et al. [34].

#### 5.3.2. Culture temperature for primary isolation

Optimal culture temperature differences exist between *F. noatunensis *subsp. *noatunsis, F. noatunensis *subsp. *orientalis *and the molluscan pathogenic strain, which probably reflect evolutionary differences related to host species and environment. Soto et al. [34] described optimal growth of *F. noatunensis *subsp. *orientalis *(putative) at 28°C, while Mikalsen et al. [13] described optimal growth of *F. noatunensis subsp. noatunensis *at 22°C. While both types of bacteria are capable of growth at 30°C, *F. noatunensis *subsp. *noatunensis *grows poorly at this temperature [19]. That *F. noatunensis *subsp. *noatunensis *was also reported as having an optimum temperature of 15-19°C and was unable to grow at 30°C [6], suggests that agar composition may be important in relation to growth at various temperatures. Kamaishi et al. [8] reported optimal growth of *Francisella *sp. from abalone at temperatures between 17 and 22°C. Suitable culture temperatures for isolation of fish and mollusc pathogenic *Francisella *would therefore generally appear to be in the range of 22°C-25°C.

### 5.4. Cell-culture

As *Francisella *spp. may be cultured on cell-free laboratory media, the benefits of culture in cell-culture may be dubious given its' technically demanding and laborious nature. However, successful cell-culture of *Francisella noatunensis *subsp. *noatunensis *has been reported in salmon head kidney (SKK-1) and Atlantic salmon kidney (ASK) cells with best growth in SHK-1 cells [6] and *Francisella *sp. (most probably *F. noatunensis *subsp. *orientalis*) isolated from tilapia was successfully cultured in chinook salmon embryo (CHSE-214) cells [2].

### 5.5. Differential phenotypical identification

*Francisella *spp. are generally rather biochemically unreactive and the number of phenotypical tests useful for differentiation of the various member species are few. Fish pathogenic *Francisella *species and *F. tularensis *share a requirement for cysteine in culture media, and the fish pathogens may thus be initially more easily confused with this species than with their phylogenetically closer relative *F. philomiragia *which grows quite happily on blood agar. While several commercial kits have been used for phenotypical profiling of fish pathogenic species, reactions may be weak and difficult to interpret [19], and published comparisons have not included *F. tularensis *or *F. novicida*. Fish pathogenic isolates may, however, be fairly rapidly differentiated from *F. tularensis *and *F. novicida *by their lack of growth on suitable media at 37°C [19,20]. Further the fish pathogenic *Francisella *may be readily distinguished from *F. philomiragia *(environmental and mammalian isolates) by their requirement for cysteine in culture media and their inability to grow at temperatures of 35°C or above and lack of production of cytochrome oxidase [13].

### 5.6. Molecular identification

#### 5.6.1. Universal PCR combined with DNA sequencing

A common theme to most, if not all initial confirmations of francisellosis, is utilisation of the polymerase chain reaction (PCR) in association with "universal" primers directed at the bacterial 16S rRNA gene. Following amplification and DNA sequencing, identification of *Francisella*-related 16S rRNA gene sequences within tissue samples allows directed culture with appropriate media. Such a strategy was used in identification of the aetiological agent of francisellosis in Atlantic cod [6,7] Atlantic salmon [1] tilapia [2,5], hybrid striped bass [37], three-lined grunt [3] and abolone [8]. Isolation and culture of the bacterium has then allowed phenotypical and genetic characterisation, which has in turn provided a basis for both phenotypical based identification and development of specific molecular assays for detection of the respective bacteria within fish tissues.

#### 5.6.2. Specific PCR and LAMP

The close relationship between the fish pathogenic *Francisella *spp. and the relatively heterogeneic *F. philomiragia *has made development of specific assays challenging. However, several more or less specific PCR assays now exist for detection of individual members of the genus *Francisella *in fish. Ottem et al. [51] described a combination of two real-time PCR assays targeting the 16S rRNA gene (detecting *F. noatunensis *subsp. *noatunensis and orientalis*) and the outer membrane protein *FopA *(detecting *F. noatunensis *subsp. *noatunensis *and *F. philomiragia*) for specific detection of either fish pathogen when used together (Table 3). More recently, a real-time PCR for specific detection of *F. noatunensis *subsp. *orientalis *with a lower detection level of approximately 25 genome equivalents has been developed [68]. Alternatives to PCR detection include the loop-mediated isothermal amplification (LAMP) reaction targeting the groEL gene of *F. noatunensis *subsp. *noatunensis*, as described by Caipang et al. [69], which has the advantage of not requiring a thermal cycler and may therefore prove suitable for use in the field. The specificity of this assay against other *Francisella *spp. was not, however, tested. The presence of an unknown number of as yet undescribed environmental *Francisella *species, including isolates apparently closely related to fish pathogenic species [12], discussed elsewhere in this review, should be borne in mind, particularly on identification of infection in novel species of fish.

**Table 3:** PCR/Real time PCR/LAMP primers (and probes) used for detection/characterisation of *Francisella *spp.

Specific for	Target gene or region	Amplicon size	Primer sequence (5' - 3')	Probe sequence (Real time PCR)	Reference
*F. noatunensis *subsp. *noatunensis *+ subsp. *orientalis*	16S *rRNA*	101 bp	**FcF50** aac-gac-tgt-taa-tac-cgc-ata-ata-tct-g	**Fc50-probe** gtg-gcc-ttt-gtg-ctg-c	Ottem et al. [51]
			**FcR50** cct-tac-cct-acc-aac-tag-cta-atc-ca		

*F. noatunensis *subsp. *noatunensis *+ *F. philomiragia*	*FopA*	85 bp	**FopAF** ggt-gcg-aac-atg-act-att-ggt-tat	**FopA-probe** ttt-gca-gtt-cag-tat-aac	Ottem et al. [51]
			**FopAR** aac-ctg-caa-ata-ctc-tac-cca-cta-act		

*F. noatunensis *subsp. *orientalis*	*iglC*	88 bp	***iglC *forward** ggg-cgt-atc-taa-gga-tgg-tat-gag	***iglC *probe** atc-tat-tga-tgg-gct-cac-aac-ttc-aca-a	Soto et al. [68]
			***iglC *reverse** agc-aca-gca-tac-agg-caa-gct-a		

*Francisella*-like	16S *rRNA*	286 bp	**FLB16S180f** gcg-gat-taa-agg-tgg-cct-ttg-c	n.a.	Hsieh et al. [42]
			**FLB16S465r** cct-gca-agc-tat-taa-ctc-aca-gg		

*Francisella *spp.	16S *rRNA*	1113 bp	**F5** cct-ttt-tga-gtt-tcg-ctc-c	n.a.	Forsmann et al. [64]
			**F11** tac-cag-ttg-gaa-acg-act-gt		

*Francisella *spp.	16S *rRNA*	~1170 bp	**Fr153F0.1** gcc-cat-ttg-agg-ggg-ata-cc	n.a.	Barns et al. [11]
			**Fr1281R0.1** gga-cta-aga-gta-cct-ttt-tga-gt		

*F. noatunensis *subsp. *noatunensis*	*groEL*	Multiple bands	**Primer set 4 (LAMP) F3** ggt-gct-caa-ata-gtt-aaa-gaa-gt	n.a.	Caipang et al. [69]
			**B3** gta-ccc-act-tgc-tcg-ata-g		
			**FIP** ttc-tgt-aag-taa-cgc-ttg-agc-taa-t**tt-tt**c-tgc-tga-tgt-agc-agg-tg		
			**BIP** aac-agg-tat-tga-taa-ggc-tgc-tg**t-ttt**-tct-gaa-caa-ggc-tta-gaa-agt		

n.a.: not applicable.

^b^: used in combination with sequencing.

#### 5.6.3. In situ hybridisation

Splettstoesser et al. [70] described fluorescent in situ hybridisation (FISH) identification and differentiation of various *Francisella *infections using probes based on the 23S rRNA molecule. This study, despite extensive comparison of non-*Francisella *strains, was limited to *F. tularensis*, *F. novicida *and *F. philomiragia *and did not, unfortunately, utilise any of the fish pathogenic species. In situ hybridisation using dioxigenin (DIG) probes directed at the 16S rRNA molecule successfully identified *Francisella *cells within many different tissue types in three-lined grunt [3] tilapia [2] and diseased cichlids [42] and most recently in farmed abalone [8] (Table 4).

**Table 4:** In situ probes used for visualisation of *Francisella *spp. in aquatic animals.

Fish type	Bacterial species	Probe type¤	Primers or probes (5' - 3')	Reference
Cichlids	*Francisella *spp.	PCR product (286bp)	**FLB16S180f** gcg-gat-taa-agg-tgg-cct-ttg-c	Hsieh et al. [42]
			**FLB16S465r** cct-gca-agc-tat-taa-ctc-aca-gg	

Tilapia	*Francisella *sp.*	PCR product (1113bp)	**F5** cct-ttt-tga-gtt-tcg-ctc-c	Hsieh et al. [2]#
			**F11** tac-cag-ttg-gaa-acg-act-gt	

Three-lined grunt	*Francisella noatunensis *subsp. *orientalis*	Antisense oligo-	**Isaki-DIG-80r** ctc-gtc-agc-atc-cga-aga-cct-gtt-a	Kamaishi et al. [3]
			**Isaki-DIG-200r** ggc-agc-gca-aag-gcc-acc-ttt-aat-ccg-cag-ata-t	

Abalone	*Francisella *sp.	Antisense oligo-	**Megai-110r** ccg-cca-ctc-gtc-agc-aag-aag-caa-gct-tct-cct-gtt-acc-gtt-cga-ctt-gc	Kamaishi et al. [8]
			**Megai-230r** cta-acg-cag-gct-cat-cca-tct-gcg-gca-gca-caa-agg-cca-cct-tta-atc-ctc-aga-tag-tat	
			**Megai-870r** gag-tac-tta-acg-cgt-tag-cta-cgc-cac-tag-atc-ctt-tac-acc-gaa-tcc-aac-agc-tag-tac	

¤ all Dioxigenin marked; * Presumptively *F. noatunensis subsp. orientalis*.

*# *primers originally published by Forsman et al. [64].

## 6. Control/treatment

Due to the intracellular location of the infecting bacteria, the normally high prevalence of infected fish, the high transmissibility and low infective dose, high morbidity and inappetance in severely infected fish, there is reason to believe that antibiotic therapy is unlikely to provide good and lasting effect on an infected population. However, Chern and Chao [33] considered a 10-14 day treatment with 30-50 mg/kg oxytetracycline as a probable effective treatment for francisellosis in tilapia, while Mauel et al. [4] and Ostland et al. [37] also reported successful treatment with tetracycline in tilapia and hybrid striped bass respectively. Minimum inhibitory concentrations (MIC) for *F. noatunensis *subsp. *noatunensis *[43] and the RLO from Taiwanese tilapia [33] are shown in Table 5.

**Table 5:** Minimal Inhibition Concentrations.

**Antibiotic (μgmL**^**-1**^**)**	*Francisella *"*philomiragia*" a.k.a. *F. noatunensis *subsp. *noatunensis*	RLO*
Florfenicol	1.0	n.d.

Flumequine	0.25	n.d.

Oxolinic acid	0.25	n.d.

Oxytet/tetracycline	0.5	1

Amoxicillin	> 64	n.d.

Chloramphenicol	n.d.	4

Erythromycin	n.d.	10

Pencillin G	n.d.	> 1000#

* (Chern and Chao [33] possibly the *Francisella*-like organism described by Hsieh et al. [2])

# units per mL.

## 7. Bacterial pathogenesis and host response

*F. tularensis*, as a serious zoonotic agent and candidate for biological warfare/terrorism is by far the most significant member of the genus in terms of human impact. While a considerable body of information relating to pathogenesis, virulence and host response (reviewed by Pechous et al. [71] is available for this bacterium, much relating to the mode of action and genetic basis for virulence remains poorly understood. Although similar work on the fish pathogenic *Francisella *species is limited, the results generated so far are generally consistent with those from studies focusing on mammalian pathogenic *Francisella *spp. Homologs of genes associated with virulence in *F. tularensis *have been identified in *F. noatunensis *subsp. *orientalis *[55], including genes (*iglA *- *D*) associated with the type 6 secretion system present on the *F. tularensis *pathogenicity island. Soto et al. [55] found that while *iglC *played no role in protection from serum killing, a functional *iglC *gene is necessary for intra-macrophage survival. Serum complement and host cell mannose receptors were also identified as important for macrophage internalisation of *F. noatunensis *subsp. *orientalis *cells. Zebrafish infected intraperitoneally with *F. noatunensis *subsp. *orientalis *displayed a tissue-specific proinflammatory response [54], with upregulation of inter-leukin-1β (highly specific to viable bacteria), gamma interferon and tumour necrosis factor alpha, 6 h post infection and lasting for up to 7 days.

## 8. Vaccination

No commercial vaccine is currently available against *Francisella *infections in fish, although several vaccine companies are involved in development work in relation to francisellosis in tilapia and cod. Development of a vaccine providing satisfactory protection toward fish pathogenic *Francisella *spp. may be challenging as observed with other intracellular bacterial pathogens such as *Renibacterium salmoninarum *and *Piscirickettsia salmonis*. Several trial vaccines against francisellosis in cod, based on simple whole-cell based preparations (bacterins) have been tested both in experimental challenges and in the field in Norway. None have yet awarded a significant or satisfactory degree of protection. Work contributing to a better understanding of immunological activity and bacterial factors involved in the disease is as yet limited, but includes characterisation of the lipopolysaccharide and β-glucan of *Francisella *"victoria" (isolated from tilapia, almost certainly *F. noatunensis *subsp. *orientalis*) [21] and identification of a strong, specific antibody response to a 20-KDa non-protein constituent (probably LPS) of *F. noatunensis *subsp. *noatunensis *in cod [72]. While a recombinant approach may, as in *P. salmonis *[73], offer the promise of increased protection, it may be worth considering the fact that no vaccine against *F. tularensis *infection in humans is as yet available [74], despite the greater knowledge of pathogen-host interactions for this disease. Rohmer et al. [75] proposed that due to the intracellular nature of these bacteria, a live (attenuated) vaccine instead of a component vaccine may be the best approach for successful vaccination. Identification of complete attenuation of *F. noatunensis *subsp. *orientalis *by mutation of the iglC* gene as described by Soto et al. [55], should provide an interesting foundation for further vaccine development.

Infection models, including intraperitoneal-, bath- and cohabitant- challenges exist for *F. noatunensis *subsp. *orientalis *[54,55] and *F. noatunensis *subsp. *noatunensis *[6,59]. Such models are an essential part in vaccine developmental work and batch testing. However, current standards for evaluation of effectiveness of fish vaccines rely on differences between relative percentage survival (RPS) in vaccinated and unvaccinated fish. This may be an effective method of evaluation of protection awarded against systemic bacterial infections normally causing acute mortality episodes e.g. various *Vibrio *infections, but may be questionable as a means of evaluating a disease like francisellosis which is normally associated (particularly in coldwater species) with a chronic infection. There is a risk that while vaccinated fish may survive the initial exposure and observation period, they may remain infected and the onset of disease merely delayed.

## 9. Concluding remarks

Despite previous recognition of the disease, the aetiological agents of francisellosis were not identified until recently. As these bacteria are not always readily observed histologically and cannot be cultured in the laboratory media used in routine fish disease investigations, it is likely that diseases caused by this group of bacteria remain under-diagnosed. Improved molecular/genetic tools for specific detection and diagnosis of francisellosis have been developed by a number of groups, but these studies are by no means complete since there remain major gaps in our understanding of the epidemiology and pathogenesis of the bacteria. We are not sure of their life cycle and the mechanisms by which they might spread in the environment. Evidence also exists for the existence of a large number of related bacteria in the environment. There is no doubt that as wild fisheries decline and our dependence on aquaculture products expands, domestication of new species will most probably result in identification of new species and strains of *Francisella *pathogenic for these species. Development of effective generic vaccines against francisellosis in fish should therefore be a research priority.

## 10. Competing interests

The authors declare that they have no competing interests.

## 11. Authors' contributions

DJC and SD both contributed to the literature review and drafting of the manuscript. Both authors read and approved the final manuscript.
